# Progress with induction of HIV broadly neutralizing antibodies in the Duke Consortia for HIV/AIDS Vaccine Development

**DOI:** 10.1097/COH.0000000000000820

**Published:** 2023-09-25

**Authors:** Barton F. Haynes, Kevin Wiehe, S. Munir Alam, Drew Weissman, Kevin O. Saunders

**Affiliations:** a Duke Human Vaccine Institute, Departments of Medicine and Immunology; b Duke Human Vaccine Institute, Department of Medicine, Duke University School of Medicine, Durham, North Carolina; c Perelman School of Medicine, University of Pennsylvania, Philadelphia, Pennsylvania; d Duke Human Vaccine Institute, Departments of Surgery, Immunology and Molecular Genetics and Microbiology, Duke University School of Medicine, Durham, North Carolina, USA

**Keywords:** B cell lineage design, broadly neutralizing antibodies, HIV vaccine, immune tolerance, improbable mutations, prefusion envelope, vaccination

## Abstract

**Purpose of review:**

Design of an HIV vaccine that can induce broadly neutralizing antibodies (bnAbs) is a major goal. However, HIV bnAbs are not readily made by the immune system. Rather HIV bnAbs are disfavored by a number of virus and host factors. The purpose of the review is to discuss recent progress made in the design and use of immunogens capable of inducing HIV bnAbs in the Duke Consortia for HIV/AIDS Vaccine Development.

**Recent findings:**

New immunogens capable of binding with high affinity to unmutated common ancestors (UCAs) of bnAb B cell lineages have been designed and strategies for stabilization of HIV Env in its prefusion state are being developed. Success is starting to be translated from preclinical studies of UCA-targeting immunogens in animals, to success of initiating bnAb lineages in humans.

**Summary:**

Recent progress has been made in both immunogen design and in achieving bnAb B cell lineage induction in animal models and now in human clinical trials. With continued progress, a practical HIV/AIDS vaccine may be possible. However, host constraints on full bnAb maturation remain as potential roadblocks for full maturation of some types of bnAbs.

## INTRODUCTION

In 2010, our group published an article “Is developing an HIV-1 vaccine possible?”, in which we noted the success in 2009 of the RV144 trial that induced nonneutralizing, antibody dependent cellular cytotoxicity (ADCC)-mediating HIV-1 antibodies that were a correlate of decreased transmission risk, and provided hope that a protective HIV vaccine was indeed possible [1]. Since that review, three HIV-1 efficacy trials, HVTN 702 (NCT02968849) [2] HVTN 705 (NCT03060629) [3] and HVTN 706 (NCT03964415) [4] have been completed that induced nonneutralizing antibodies and generated a variety of fragment crystallization (Fc) region receptor (R)-mediated antiviral responses, yet these trials failed to show protection from HIV-1 transmission. Recently, the finding in the HVTN 703/HPTN 081 (NCT02568215) and HVTN 704/HPTN 085 (NCT02716675) clinical trials in which passive administration of the CD4 binding site (CD4BS) broadly neutralizing antibody (bnAb) VRC01 was able to protect against HIV-1 strains that were sensitive to the bnAb, suggested that potent and high titers of bnAbs could indeed protect against HIV-1 transmission [5^▪▪^]. Thus, the main focus of the HIV-1 vaccine field now is to induce HIV-1 bnAbs [6^▪^], and if possible, combine such a vaccine with an immunogen that can induce anti-HIV-1 CD8 T cell responses to decrease the levels of bnAbs needed for protection [7^▪^]. Here we review progress made by the NIAID-funded Consortia for HIV/AIDS Vaccine Development (CHAVD) at Duke University in the effort to induce HIV-1 bnAbs with a vaccine. 

**Box 1:**
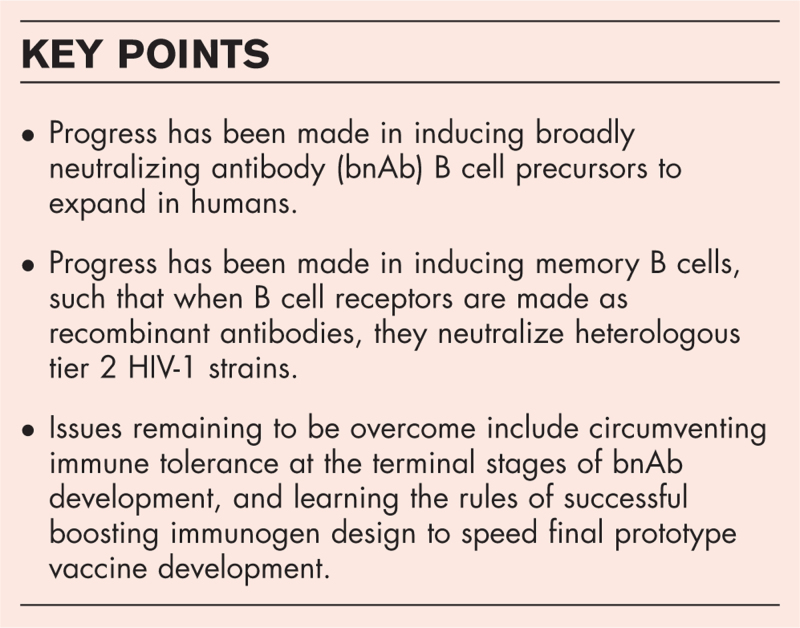
no caption available

## WHY BROADLY NEUTRALIZING ANTIBODIES ARE DIFFICULT TO INDUCE

BnAbs are difficult to induce both because of characteristics of the HIV-1 envelope (Env) and because of host controls on induction of bnAb B cell precursors [6^▪^]. As with many viruses with fusogenic surface molecules, the HIV-1 Env undergoes extraordinary receptor-mediated conformational changes. The prefusion state of Env trimer is held in dynamic equilibrium between “closed” conformations in which the Env protomers are tightly packed together to more “open” conformations in which the Env protomers are spread farther apart, with “open” conformations capable of being induced and stabilized by CD4 binding [8–11]. While many nonneutralizing immunodominant antibody epitopes are present on “open” Env forms, bnAb epitopes are the predominant antibody epitopes on “closed” prefusion conformations. Thus, when immunizations have been carried out with more open Envs, the dominant antibody responses elicited have been to nonneutralizing epitopes [6^▪^,^12^]. While immunization with a prefusion Env form is desirable, to date, configuration of a definite and stable prefusion envelope has not yet been achieved [8,9,12,13]. On the prefusion Env, bnAb epitopes are transiently occluded by a dense glycan shield [14], such that most bnAbs need to either bind to or accommodate glycans to engage a protein bnAb epitope [15]. Since Env glycans are modified by host glycosyltransferases and glycosidases they are similar to glycans on host proteins, suggesting that to respond to many bnAb epitopes [16], the immune system must mediate an antiglycan, autoantibody response. A similar conundrum exists with antibodies against the bnAb epitopes on the gp41 membrane proximal external region (MPER) in that for MPER bnAbs to neutralize HIV-1, they must not only bind the neutralizing bnAb polypeptide epitope, but also must bind to the virion lipid membrane which comes from host CD4+ cells during virion budding [17–20]. Moreover, the proximal MPER bnAb epitope has the sequence ELDKWA that is shared with the host tryptophan enzyme kynureninase [21,22]. Thus, it has been proposed that immune tolerance controls such as anergy, can limit bnAb induction because the immune system sees some bnAb epitopes as autoantigens [21–23]. However, a strong adjuvant in mice can overcome peripheral anergy and induce bnAb production in bnAb knock-in mice [24].

Our group and others have found many immune system perturbations in HIV-1-infected individuals who made bnAbs compared to those that did not, which included HIV-1 infection-induced loss of CD4^+^ T regulatory cells [25], decreased NK suppression of CD4^+^ T follicular helper cells (TFH) [26], an expanded autoreactive B cell repertoire [27^▪^] and a higher circulating memory TFH frequency [25,28]. All of these immune perturbations lead to an immune microenvironment that is permissive for bnAb development [25,27^▪^].

Another limiting factor in the induction of bnAb B cell lineages is that they have an excess of mutations that are not typically generated by the somatic hypermutation machinery in conventional antibody responses [29]. These improbable mutations can play key roles in conferring neutralization breadth and are required for bnAb development [29]. Thus, design of immunogens through a process termed mutation guided immunogen design that can select for such improbable functional bnAb mutations is critical for bnAb boosting immunogen designs [29,30]. The natural circulating variability in key epitopes of interest also fundamentally limits breadth, and iterative boost designs based on defining Env key resistance signatures inspired by recapitulating antibody evolution of breadth *in vivo*[31] may further advance the development of breadth and potency of bnAb cross-reactivity to heterologous viruses. This could be used as a complementary strategy to selection of improbable mutations for guiding immunogen design, with the potential to further enhance the breadth of vaccine responses during antibody maturation.

Finally, a vaccine that simultaneously elicits bnAbs to multiple epitopes will likely be needed to prevent virus escape [6^▪^]. A particularly challenging aspect of elicitation of multiple bnAbs is that many bnAbs require long heavy chain complementarity determining regions (HCDR3s) to accommodate glycans or bind lipids at or near Env bnAb epitopes. However, long HCDR3 antibodies are disfavored in the human B cell repertoire and thus the precursors of V2-glycan, V3-glycan and some MPER bnAbs are rare since many B cells with heavy chain variable region (VH) genes with long HCDR3 regions can be eliminated early in B cell development.

## PROGRESS IN BROADLY NEUTRALIZING ANTIBODY IMMUNOGEN DESIGN

The biology of bnAbs has necessitated the use of structural immunogen design [32], germline or UCA-targeting immunogen design [33,34,35] and B cell lineage [33] and mutation-guided immunogen [29,30] designs to make progress in vaccine designs for inducing bnAbs [6^▪^]. Immunofocusing strategies have induced B cell lineages producing neutralizing antibodies targeted to the HIV fusion peptide [36].

Recent progress has been made in stabilizing the HIV-1 envelope in more closed conformations to be more near the prefusion state of Env. Sanders and Moore recently drew attention to their use of proline mutations to stabilize soluble Env trimers (SOSIP) and how similar proline mutations were useful in the stabilization of the SARS-CoV-2 spike trimer [37]. Wrapp *et al.* have further stabilized the Env trimer with additional prolines at aa 568 and 569 in addition to the original proline at 559 in the SOSIP trimer [38^▪^]. While not completely stabilizing the trimer in a fully prefusion state, such mutations, as well as those of others [39,40] have greatly stabilized HIV-1 Env for vaccine trials. Even when not fully stabilized, immunization of macaques with soluble SOSIP trimers can induce potent autologous, though not heterologous, tier 2 neutralizing antibodies [41,42,43^▪^,^44^]. New technologies for studying Env conformations such as time-resolved, temperature-jump small angle X-ray scattering to monitor structural rearrangements in an HIV-1 Env ectodomain construct with microsecond precision are now being used to determine novel ways to stabilize Env for vaccine immunogens [45].

Henderson, Acharya *et al.* have studied the ontogeny of one V3-glycan directed bnAb lineage, called DH270, and generated ten cryoEM maps to identify bottlenecks along the affinity maturation pathway [46^▪▪^]. Wiehe *et al.* have combined structure and mutational-based immunogen design to achieve successful prime and boost results in V3-glycan bnAb unmutated common ancestor (UCA) human antibody VH + VL gene knock-in mice, thus beginning to learn the rules for bnAb prime and boost designs [30]. For CD4 binding site (CD4bs) antibodies, Sanders *et al.* demonstrated the selection of VRC01 class antibody precursors with insertion and deletion mutations required for bnAb activity in CH31 UCA antibody knock-in mice, though no general rules were apparent for how to achieve this selection routinely [47]. It has been proposed that creation of an affinity gradient between antibody intermediate states along a bnAb maturation pathway is one rule of sequential immunogen design [30,33,48]. Zhang *et al.* demonstrated in 2016 that a critical feature of induction of HIV-1 heterologous neutralizing antibodies in macaques was selection of antibody mutations required for a fast bnAb association rate for Env [49]. Hossain *et al.* have now presented data that support a kinetic model for B cell activation in which Env protein affinity discrimination is based not on overall *K*_D_ but rather on sensing of association rate and a threshold antigen-BCR half-life [50]. Roark, Shaw *et al.* have developed a simian-human immunodeficiency virus (SHIV) infection model in rhesus macaques with bnAbs identified in ∼15% of infected monkeys [51]. More recently, Alt *et al.* have developed humanized V(D)J-rearranging and TdT-expressing mouse vaccine models with physiologic levels of HIV-1 bnAb precursors [52]. Thus, progress is being made on developing powerful animal models for learning the rules for inducing bnAbs.

In addition to immunogen design, progress has been made in new adjuvant development. Empty ionizable lipid nanoparticles have been demonstrated to be potent adjuvants for protein immunogens and to induce a number of cytokines and chemokines that promote TFH cell and antibody development [53^▪^]. Because bnAbs have high levels of improbable somatic mutations, a successful HIV vaccine will be required to induce persistent B cell germinal center responses such that memory B cell receptors can acquire the rare mutations needed for full B cell maturation. Lee *et al.* have demonstrated induction of germinal centers with long duration in nonhuman primates with the adjuvant saponin/MPLA nanoparticles (SMNP) formulated with an Env trimer [54^▪^].

## MODIFIED mRNA/LIPID NANOPARTICLE HIV VACCINE PLATFORM

**FIGURE 1:**
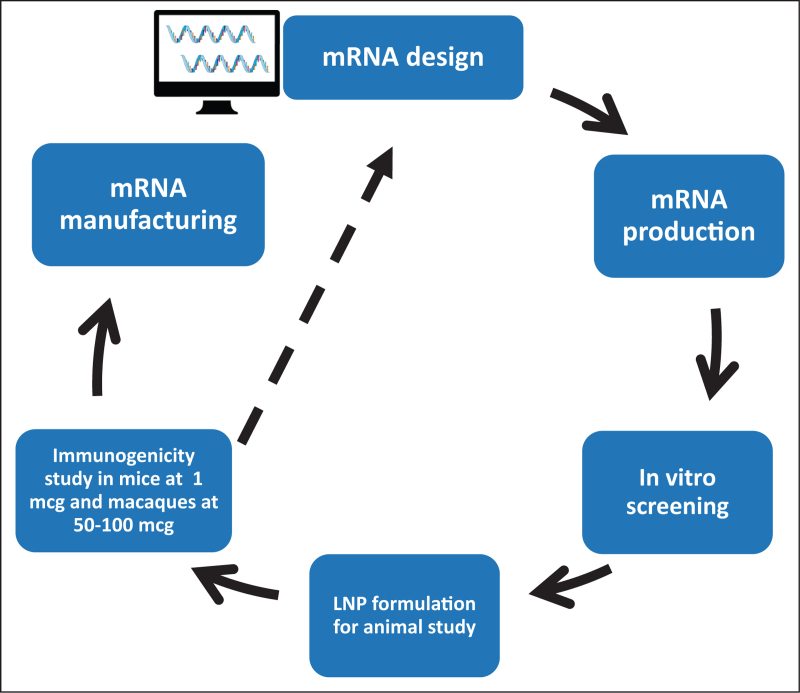
Modified mRNA HIV-1 vaccine immunogen design strategy. Modified mRNAs are designed and produced for screening for adequate in vitro expression, followed by encapsulation in ionizable LNPs for in vivo immunization studies. If immunogenicity is acceptable at appropriate doses in mice or macaques, the mRNA can be down-selected for manufacture. If immunogenicity is not acceptable, redesign of the mRNA is necessary (dotted line). LNPs, lipid nanoparticles.

Modified mRNA encapsulated in ionizable lipid nanoparticles (LNP) was a powerful platform for accelerating development of COVID-19 vaccines [55,56]. Modified mRNA vaccines have started to be used in preclinical studies as well as in clinical trials of HIV-1 vaccine candidates. The manufacture of mRNA/LNP vaccines is rapid, and generally, the cost is lower than for protein vaccines [57]. However, for mRNA down-selection, stabilization mutations are necessary to ensure a high percentage of well folded Env following mRNA expression [58^▪▪^] and testing for immunogenicity at appropriate doses in animal models is critical to ensure adequate *in vivo* expression (Fig. 1). Two studies have recently demonstrated in UCA antibody knock-in mice that mRNA-encoded membrane anchored trimers may have advantages over protein vaccines. Melzi *et al.* suggested that mRNAs may lower the threshold to activate HIV Env V2 apex-directed broadly neutralizing B cell precursors in humanized mice [59]. Wiehe *et al.* demonstrated that mRNA/LNP immunogens can select for improbable mutations necessary for bnAb recognition of Env glycans better than protein immunogens [30].

## PROGRESS IN DUKE THE CONSORTIA FOR HIV/AIDS VACCINE DEVELOPMENT IN HUMAN CLINICAL TRIALS

**FIGURE 2:**
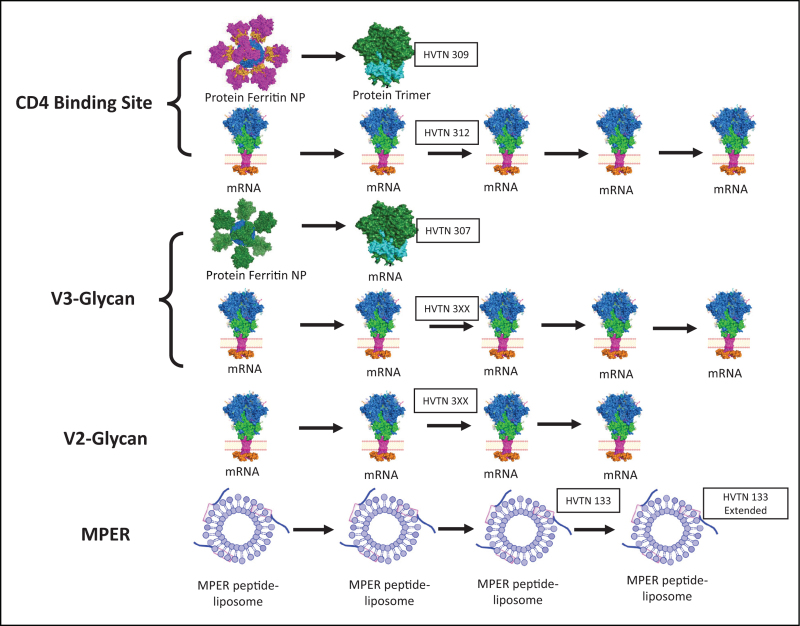
The Duke CHAVD Clinical Trials Planned Portfolio. For CD4BS bnAb induction, we have two clinical trials planned, one with protein (HVTN 309) and one as mRNA/LNPs encoding transmembrane stabilized gp160 Envs (HVTN 312). Both the protein and the mRNA/LNP versions are of the VH1–46-type of CD4 mimicking CD4BS bnAb designed for the CH235 bnAb lineage. For V3-glycan bnAb induction, again, we have two clinical trials planned, one as protein (HVTN 307) and one as mRNA/LNP. Since a key limiting factor in inducing V3-glycan bnAbs is the dearth of bnAb precursors of this type, we have designed a priming immunogen that binds to three different V3 glycan lineage UCAs. Similarly, for V2-glycan bnAb induction, mRNAs are being designed that bind to multiple UCAs. Finally for MPER bnAb induction, in the HVTN 133 clinical trial we have used repetitive immunizations with an MPER peptide-liposome formulation. bnAb, broadly neutralizing antibody, LNP, lipid nanoparticles; UCA, unmutated common ancestor.

Figure 2 outlines the Duke CHAVD planned portfolio of immunogens to be developed by 2026. Table 1 shows the HIV Vaccine Trials Network (HVTN) trials of the Duke CHAVD that are ongoing or planned. The HVTN 115 trial (NCT03220724) was designed based on the first antibody-virus co-evolution study in 2013 [60] and has just finished enrollment. It is based on the observed sequential Env evolution that occurred as the CH103 CD4BS bnAb lineage evolved in the CH505 HIV-1-infected subject. Interestingly, when the CH103 lineage acquired bnAb maximum breadth, mature bnAbs also acquired autoreactivity with host antigens [60]. When we used this regimen in rhesus macaques and CH103 UCA knock-in mice, we found a number of immune tolerance mechanisms that limited this type of CD4BS bnAb development [61]. The HVTN 115 trial tests whether a CH505 transmitted/founder (TF) gp120 Env priming immunogen can expand CH103 UCAs and if subsequent sequential Env boosting immunogens can induce affinity-matured bnAbs [61]. The results of the immunogenicity studies should be available early in 2024. The HVTN 135 trial (NCT04607408) is a similar study but with only the CH505 TF gp120 administered as a repetitive priming immunogen in African infants born to HIV+ mothers. The HVTN 300 trial (NCT04915768) is also nearing completion and studies a stabilized CH505 SOSIP trimer that is a high affinity targeting Env for the CH103 UCA formulated with the TLR-7,8 agonist, 3M052, that protected monkeys from autologous SHIV CH505 challenge [43^▪^].

**Table 1:** The Duke Consortia for HIV/AIDS Vaccine Development clinical trials

Protocol #	Regimen	bnAb strategy	Status	Research questions
HVTN 115	EnvSeq-1 (CH505TF, CH505w53, CH505w78, gp120s) and CH505 M5 gp120 Envs with GLA-SE adjuvant	Unmutated common ancestor (UCA)-targeting, Lineage-based vaccine design: CD4bs CH103 lineage + CH235 lineage	Opened: Oct 2017 Near completion	•NAbs elicited by CH505TF gp120 adj. with GLA-SE (dose-finding)? •NAbs elicited by 3 EnvSeq-1 immunogens administered in sequential and additive approaches and CH505 M5 gp120 protein administered as a single immunogen (all GLA-SE adjuvant)?
HVTN 133 and HVTN 133 Extended	gp41 MPER-656 liposome with Alum adjuvant (HVTN 133); HVTN 133 Extended − boosts with mRNA/LNP that encode a transmembrane gp41 fragment with full MPER	Unmutated ancestor-targeting, Epitope-based vaccine design	Original trial opened: August 2019 Enrollment closed; Follow-up completed Induced B cells producing heterologous neutralizing antibodies; Stopped after 2–3 immunizations due to one event of presumed polyethylene glycol anaphylaxis	•Does MPER peptide elicit 2F5-like NAbs in humans? •Will this inform the pathway to B-cell lineage development of bNAbs? •Does an mRNA encoding a TM-MPER Env boost and affinity mature existing heterologous neutralizing bnAbs to greater breadth and potency?
HVTN 135	CH505 gp120 TF with GLA-SE adjuvant	UCA-targeting: CD4bs CH103 lineage	Opened: October 2020	•Does vaccination of healthy, HIV-exposed uninfected infants with CH505TF gp120 adj. with GLA-SE initiate B-cell lineages potentially capable of generating a broadly neutralizing antibody response?
HVTN 300	CH505 TF chTrimer with 3M-052/Alum ^∗^Part B: lower dose 3M-052/alum & 3M-052 w/o alum	UCA-targeting CD4bs CH103 lineage	Opened: June 2021 Enrollment completed Part B: Study of 3M052 adjuvant lower dose with and without Alum	•Does CH505 TF chTrimer + 3M-052/Alum elicit: •CD4 binding-site, CH505TF-specific memory B cells? •Autologous tier 2 nAbs? •Parts B & C: Does 3M-052 without alum improve neutralization & reduce reactogenicity?
HVTN 307	V3G CH848 Pr-NP1 (protein NP prime) + V3G CH848 mRNA-Tr2 (mRNA trimer boost) + 3M-052+Alum adjuvant	V3-glycan bnAb B cell DH270 lineage	Clinical trial to began August 2023	•Does V3G CH848 Pr-NP1 prime expand DH270-like B cell precursors? •How far down the maturation pathway of DH270 B cell lineage does the V3G CH848 Pr-NP1 prime and the V3G CH848 mRNA-Tr2 boost stimulate? •Can human-host controls that constrain this type of V3 lineage be detected in this human clinical trial? If yes, to what degree and by what mechanisms?
HVTN 309	CD4bs CH505M5 Pr-NP1 (prime) + CD4bs CH505TF Pr-Tr2 (boost) +3M052 + Alum Adjuvant or empty LNPs	Prime and boost VH1–46-type of CD4 mimicking, broad and potent CD4BS bnAbs	Clinical trial to begin March 2014	•Can these proteins prime and boost VH1–46 type of CD4BS bnAbs and select for improbable mutations needed for bnAb maturation?
HVTN 312	CH505 M5 G458Y N917D gp160 mRNA/LNP prime; CH505 TF gp160 boost mRNA/LNP boost	Prime and boost VH1–46-type of CD4 mimicking, broad and potent CD4BS bnAbs	Clinical trial to begin May 2024	•Can the mRNA versions of immunogens used in HVTN 309 prime and boost as well as the protein versions of these immunogens in HVTN 309?
HVTN 3XX	CH848 gp160 mRNA/LNP as a prime, CH848 10.17 gp160 boost	Prime designed to induce multiple types of V3-glycan bnAb lineages	Clinical trial to begin September 2024	•Can this immunogen prime for multiple V3-glycan bnAb B cell lineages and address the problem of paucity of V3-glycan bnAb precursors?

bnAb, broadly neutralizing antibody; LNP, lipid nanoparticle; MPER, membrane proximal external region; UCA, unmutated common ancestor.

A more potent type of CD4 mimicking, CD4bs bnAb that exclusively uses the VH1–46 gene segment appears to be less controlled by tolerance mechanisms, and unlike the VRC01 class of CD4bs bnAbs, does not require rare light chain features such as deletional mutations and short LCDR3s for neutralization breadth. We have shown that VH1–46 CD4bs bnAbs can be expanded in UCA knock-in mice and macaques by the CH505 M5, G458Y stabilized SOSIP trimer with homogeneous man5 glycans [44,62,63]. The HVTN 309 and HVTN 312 clinical trials will test as proteins or mRNA/LNPs, respectively, the first two prime and boost immunogens for eliciting this type of CD4bs bnAbs (Fig. 2, Table 1).

For V3-glycan induction, the HVTN 307 trial and HVTN 3XX (trial to be named) will similarly study protein prime, mRNA/LNP boost versus mRNA/LNP prime and boosts, respectively. For the protein primes we are using ferritin-Env multimer nanoparticles based on recent data that nanoparticles are advantageous immunogens compared to soluble trimers [64].

To overcome the dearth of V2-glycan bnAb precursors, Hahn *et al.* have used neutralization signature analysis [65] to define Envs that can bind and trigger multiple V2 glycan UCAs [66]. They have begun to use such Envs to explore the types of V2-directed antibodies they induce [66]. Hahn and team have also defined simian immunodeficiency viruses that induce Env V2 targeted responses that may be used as boosts for V2 UCAs expanded by HIV Envs [67,68]. The best of V2-glycan UCA targeting Envs will be available for down-selection in Qtr. 4, 2023.

Finally, our greatest success to date in HIV-1 clinical trials has come with the gp41 MPER peptide-liposome in the HVTN 133 clinical trial (NCT03934541) (Fig. 2, Table 1). This work had its beginning with the observation in 2005 that the two prototype MPER bnAbs 2F5 and 4E10 bound both to gp41 MPER and bound to lipids, implying that vaccine induction of such antibodies would need to be directed to both lipids and gp41 protein epitopes [20]. Later studies demonstrated that MPER antibodies required lipid reactivity to neutralize HIV-1, by tethering the MPER antibodies to the viral membrane [17,18,69]. Thus, we designed an MPER peptide-liposome that contained both lipids and the gp41 MPER bnAb epitopes [70]. Moreover, immunization of rhesus macaques with this immunogen initiated MPER bnAb epitope-targeted antibodies [49].

**FIGURE 3:**
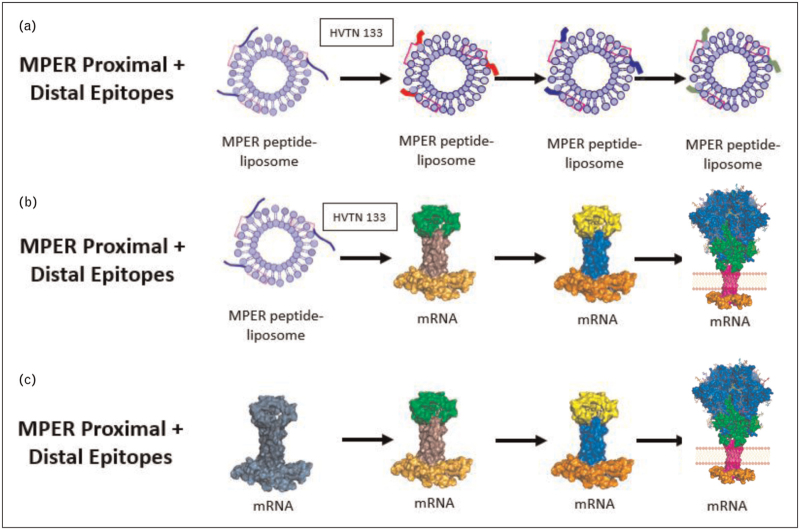
Immunogen combinations undergoing testing for induction of proximal and distal gp41 bnAbs. Panel a shows the MPER peptide-liposome used in HVTN 133 that induced heterologous neutralizing antibodies targeting the proximal MPER region. Boosts such priming have been designed to trigger both proximal and distal MPER epitopes. Panel b shows the HVTN 133 prime immunogen with boosts of mRNAs designed as transmembrane expressing modified mRNAs or transmembrane gp150  or gp160 trimers. Panel c shows the same boosts being primed by mRNAs that encode a protein or proteins that recognize both proximal and distal MPER bnAb precursors. bnAb, broadly neutralizing antibody; MPER, membrane proximal external region.

In the HVTN 133 trial, we found that three immunizations of the MPER peptide liposome induced not only a polyclonal expansion of bnAb precursor B cell lineages, but also selected for mutant antibodies of these memory B cell lineages that had affinity matured to the point of achieving heterologous tier 2 HIV-1 neutralization [71^▪▪^]. The next step is to design boosting immunogens to induce greater breadth and neutralization potency in such bnAb B cell lineages (Fig. 3).

Finally, for a successful prototype vaccine, our goal is to induce broad and potent bnAbs with several of the sequential immunogen sets shown in Fig. 2. Only by inducing more than one specificity of bnAb can we hope to attain the breadth and potency of neutralization necessary for prevention of HIV-1 transmission at mucosal sites.

## CONCLUSION

Success of induction of CD4bs precursors in humans in the IAVI G001 clinical trial [72^▪▪^] and success in inducing both MPER bnAb precursors and mature heterologous neutralizing antibodies in humans in the HVTN 133 clinical trial [71^▪▪^] have energized the HIV vaccine development field and provided hope that a vaccine can indeed be made. Ultimately success will only come, however, if more can be learned about the specific rules of how to best optimally design sequential boosting immunogens that can select for bnAb functional improbable mutations to enable the induction of antibodies with a capacity to recognize HIV-1 diversity. Moreover, overcoming anergy and other tolerance controls of disfavored B cells with maturing bnAb B cell receptors will be critically important. Importantly, work demonstrating strong adjuvants can overcome peripheral tolerance for MPER bnAb induction emphasizes the importance of adjuvant research in HIV vaccine development [24]. Finally, induction of high titers of long-lasting plasma and mucosal bnAbs that will not require frequent boosting to maintain efficacy will be critical for a successful HIV vaccine [5^▪▪^].

## Acknowledgements

*The authors acknowledge the discussions and contributions of the CHAVD Scientific Leadership Group members Beatrice Hahn, George Shaw, Frederick Alt, Kshitij Wagh, Garnett Kelsoe, Bette Korber, Andrew McMichael and Persephone Borrow. And contributions from Stuart Shapiro and Kelly Cuttle. The CHAVD Team is grateful for the support and partnership of the Division of AIDS and the HIV vaccine trials network.*

### Financial support and sponsorship

*Supported by the NIH, NIAID, Division of AIDS grant UM1-AI144371 for the Consortia for HIV/AIDS Vaccine Development.*

### Conflicts of interest

*B.F.H., K.W., S.M.A., D.W. and K.O.S. each have patents submitted on components of the Duke CHAVD portfolio discussed in this review.*

## REFERENCES AND RECOMMENDED READING

Papers of particular interest, published within the annual period of review, have been highlighted as:

▪ of special interest
▪▪ of outstanding interest
